# The Ecological-Enactive Model of Disability: Why Disability Does Not Entail Pathological Embodiment

**DOI:** 10.3389/fpsyg.2020.01162

**Published:** 2020-06-11

**Authors:** Juan Toro, Julian Kiverstein, Erik Rietveld

**Affiliations:** ^1^Center for Subjectivity Research, Faculty of Humanities, University of Copenhagen, Copenhagen, Denmark; ^2^The Enactlab, Copenhagen, Denmark; ^3^Amsterdam Brain and Cognition, Amsterdam, Netherlands; ^4^Amsterdam University Medical Center, Amsterdam, Netherlands; ^5^Department of Philosophy, University of Twente, Enschede, Netherlands; ^6^Institute for Logic, Language and Computation, Faculty of Science, University of Amsterdam, Amsterdam, Netherlands

**Keywords:** disability, medical model, ecological psychology, enactive cognitive science, normality, lived body, affordances, pathology

## Abstract

In the last 50 years, discussions of how to understand disability have been dominated by the medical and social models. Paradoxically, both models overlook the disabled person’s experience of the lived body, thus reducing the body of the disabled person to a physiological body. In this article we introduce what we call the Ecological-Enactive (EE) model of disability. The EE-model combines ideas from enactive cognitive science and ecological psychology with the aim of doing justice simultaneously to the lived experience of being disabled, and the physiological dimensions of disability. More specifically, we put the EE model to work to disentangle the concepts of disability and pathology. We locate the difference between pathological and normal forms of embodiment in the person’s capacity to adapt to changes in the environment. To ensure that our discussion remains in contact with lived experience, we draw upon phenomenological interviews we have carried out with people with Cerebral Palsy.

## Introduction

According to the influential but widely criticized medical model, disability can be understood in terms of functional limitations of a disabled person’s body caused by a clinically observable pathological condition. Disability is something to be diagnosed, treated and cured through rehabilitation or normalization (cf. Moore and Slee, 2012, p. 228). Many theorists in the field of disability studies claim however, that this conception of disability as an individual pathology is the outcome of a medicalization of physical impairments that mistakenly locates the disability within the body of the individual person taken in isolation from the social world (Oliver, 1996; Thomas, 2007; Beresford, 2012). In the last years, disability movements in the United Kingdom and North America have emphasized the social situation of disabled people and the way in which disabled people are excluded or stigmatized because of their different forms of embodiment (see McRuer, 2006; Shildrick, 2009; Davis, 2017). The social model claims that disability is not an individual physical condition, but is the outcome of “socially produced inequality and dependency” (Beauchamp-Pryor, 2012, p. 178). Disability so-conceived is a social category: a means of classifying and treating people in ways that lead to discrimination and oppression comparable to that experienced by ethnic minorities (see UPIAS, 1976; Shakespeare, 2006).

Both the medical and social models of disability are premised on concepts of embodiment that fail to adequately recognize how disabled bodies are lived bodies that have their own first-person perspective on the world. The medical model understands disability in terms of the body of the disabled person as described objectively and scientifically. At the same time this model of disability fails to recognize the lived embodiment of disabled persons^1^. The social model is arguably guilty of a similar neglect of the embodied lived experience of the disabled person. In distinguishing disability from physical bodily impairment, the social model leaves in place a medicalized understanding of the disabled person’s bodily impairment. The social model has good reasons for foregrounding the marginalization, exclusion and oppression of disabled people from full participation in wider society. However, such a focus threatens to eclipse attention to how the disabled person’s lived experience of the world is shaped by their bodily impairment (Shakespeare, 2006; Scully, 2008; Beaudry, 2016). In line with the social model, we will question the medical model’s conflation of disability with the impairment of the physical body of disabled persons. Nevertheless, unlike the social model, we do so on the basis of how the disabled person experiences the world through their embodiment in it.

We propose a model of disability which we will call *the Ecological-Enactive (EE) model of disability*, that takes into account the valuable contributions of both the medical and the social model, without being reducible to either of them. The EE model draws upon enactive cognitive science to offer an account of how a person’s body can be both a *lived body* that has its own first-person perspective on a meaningful world, and at the same time a *living body* whose biological organization can be understood and explained from a third-person scientific perspective (Thompson, 2007; Di Paolo et al., 2017; Gallagher, 2017). From ecological psychology we borrow the conception of the environment as furnishing *affordances* – possibilities for action the person can make use of because of the bodily skills and abilities they have developed (Gibson, 1979; Stoffregen, 2003; Chemero, 2009; Rietveld and Kiverstein, 2014). The EE model proposes to understand disability in terms of a person’s embodied skills for responding to the affordances of their environment^2^.

The EE model of disability aims to do justice to how a disabled person experiences the world through the medium of the lived body. At the same time the EE model aims to integrate such a first-person account of the difference in embodiment the disabled person can experience with a third person perspective on embodiment. In doing so it avoids pathologizing the disabled person’s living body.

To ensure our discussion remains in contact with lived experience, we draw upon interviews and experiments we have carried out with people with Cerebral Palsy (CP). CP is an umbrella term covering “a group of disorders affecting the development of postural and motor control and occurring as a result of a non-progressive lesion in the developing central nervous system, causing activity limitation” (Bax et al., 2005; see also Rosenbaum et al., 2007). CP is an especially interesting disability for our purposes, since it disrupts the often taken for granted control a person exerts on their body. The “magic” that seems to link the person’s decisions to their bodily movements is disturbed, generating in the person a different way of being-in-the-world (Merleau-Ponty, 2012).

We conducted an experiment in which people with CP interacted separately with a stranger, a relative, and a physiotherapist. They were given six simple tasks to perform: (1) shaking hands, (2) passing and receiving an empty cup, (3) passing and receiving a cup with water, (4) passing and receiving a small coin-shaped object, (5) playing patty-cakes, and (6) lifting up a tray with a cup of water on it.^3^ We recruited the participants in collaboration with a healthcare institute working with CP. The participants were chosen according to their location and degree of CP, so that all participants with CP (*n* = 11) had limited bodily functionality in one or both hands. The participants with CP correlated with level I to III in the gross motor classification system (GMFCS) (Palisano et al., 1997)^4^.

Here, we will draw upon the phenomenological interviews we carried out with the participants following their performance of the tasks.^5^ Phenomenological interviews are second person semi-structured interviews that use open ‘how’ questions, intended to make the participants aware of specific, phenomenologically relevant aspects of their experience. We describe phenomenological interviews as “second-person” to emphasize how the interview is open-ended. The knowledge coming out of the interview is the joint product of the intersubjective interaction of the interviewer and the interviewee. Among the aspects that participants reflect on are how they experienced their own bodies, the other person, the level of difficulty of the task, and so on.^6^ In these interviews, of around 20 min each, we find that many people with CP are able to adapt to the challenges that arise in their practical engagement with the environment just as well as people that are not disabled. Not all people with CP experience a pathological form of embodiment. Disability should not be conflated with pathology. These phenomenological reports will be complemented throughout this article with reports found in the literature of disabilities and pathologies, in order to make evident important contrasts between pathological and non-pathological forms of embodiment.

Our article will be divided into six parts. In section “The Medical and Social Models of Disability” we offer a brief overview of the medical and social models of disability. We show how both models of disability risk pathologizing the embodiment of disabled persons by understanding the embodiment of the disabled person solely in terms of physical impairment. In section “The Embodiment of Disability” we provide an ecological and enactive account of the lived body in terms of the dynamics of the living body’s selective engagement with a landscape of affordances (Rietveld and Kiverstein, 2014). Section “Normal and Pathological Embodiment: Toward an Ecological-Enactive Model of Disability” puts this account to work to understand the difference between healthy or normal, and impaired or pathological forms of embodiment. We use our ecological-enactive model to question an understanding of the bodily restrictions and limitations of the disabled person in terms of impairment. In section “Pathological and Normal Embodiment in People with Cerebral Palsy” we offer contrasting reports from people with CP we take to illustrate this difference. Some of these reports, we suggest, are best interpreted as indicating pathological embodiment, while others are illustrative of how CP does not necessarily lead to pathological embodiment. Section “Normality as optimality and the tendency toward an optimal grip” deepens our claim that a person with disabilities can nevertheless be considered to be normally embodied. Our article closes in section “How to understand the “dis” in “disability”?” by reflecting on the question of how to understand the ‘dis’ in disability. In our article we stress what disabled people are still able to do in their everyday engagement with the world, but we in no way wish to downplay the severity of the daily challenges they face. Despite the limitations they experience in their own abilities, and their vulnerability, disabled people are often skillfully able to find a way to the affordances needed to meet these daily challenges.

## The Medical and Social Models of Disability

A model of disability aims on the one hand to account for what it means to be disabled, and on the other to identify the causes of disability (Silvers, 2010). A model should identify for instance why it is that a person experiences the limitations associated with disability. The medical and social models have returned competing answers to this question. The medical model has tended to emphasize biological defect and dysfunction in answering the question of what disability is. On this model the limitations disabled people experience are accounted for by reference to some biological pathology – a clinically observable impairment in bodily structure or function (Boorse, 2010). The medical model recognizes that functional limitations are dependent on a myriad of environmental factors. However, disability is understood as essentially a health problem that requires medical treatment aimed at enabling disabled persons to adjust to society^7^.

The social model by contrast understands the limitations disabled people experience in terms of their social isolation, oppression and exclusion from participation in social life. Its proponents have distinguished *impairment* as understood in the medical model as a natural, biological fact, from *disability* conceived of as an artificial social classification (see Barnes, 2012). The limitations disabled people experience are caused by factors that come from outside of the person, not from their impairment. The real problems disabled people face come from “the surrounding social, institutional, and physical environment with which persons with disabilities must deal” (Asch and Wasserman, 2006, p. 166).

We agree with the social model that it is important to disentangle impairment from disability. A model of disability should, at minimum, account for the difference between bodily impairments that are normal, and those that are disabling. Everyone is impaired with respect to some functions (Boorse, 2010). People are unable to see ultraviolet light for instance, or swim across the Pacific Ocean. These examples of limitations in a person’s abilities do not count as disabilities because they are within the range of what is considered normal. What makes the difference between an impairment that is classified as normal, and one that is agreed to be disabling of a person?

The question of what it means to be normally embodied is central to understanding disability. As Davis notes in his introduction to the *Disability Studies Reader*: “To understand the disabled body, one must return to the concept of the norm, of the normal body” (c.f. McRuer, 2006; Davis, 2017, p. 16). According to Davis, the concept of normality as we use it today has a relatively recent history. It first emerged alongside the field of statistics in the middle of the 18th century. Notions like average and standard deviation were initially applied to astronomical observations, but they were applied to the human body in the work of Adolphe Quetelet (see Canguilhem, 1991/2015, p. 154–159; Davis, 2017). By identifying the average with the normal, the physiologist could determine objectively (i.e., quantitatively) whether a specific function or parameter such as height, weight, intelligence or strength was normal or deviant. Furthermore, based on such a statistical conception of normality, a ranking could be formed from what is normal in a population to what is above or below average. Variations from what is normal can be either good and socially desirable – better than average intelligence – or bad and undesirable – a physical defect or disease to be treated and cured. As Davis notes: “When we think of bodies, in a society where the concept of the norm is operative, then people with disabilities will be thought of as deviants.” (Davis, 2017, p. 17).

Disabled people clearly deviate from what is the average or typical body. The person with CP will differ in their movement capacities from the average person, but such a restriction in their movement capacities shouldn’t, we believe, be taken to entail that they are pathologically embodied. Thus consider as one example how *CN*, one of the participants in our experiment, with spastic CP that affects her left arm and hand, describes her experience of horse riding:

“*It’s dangerous doing this specific thing with the horse, because it demands two hands, but I don’t think about not having two hands. And then I get mad because my dad is overprotective, because I can easily do it*.”

The inference from statistical deviance to pathological embodiment fails to take into account the lived experience of the person. CN’s experience of horse riding is that she can do things with the horse that a non-disabled person would typically do with two hands. Her embodied experience of the horse is comparable to that of any non-disabled person that knows how to ride a horse. CN may not share all of the movement capabilities of people that do not have CP but this difference does not license the conclusion that she is pathologically embodied.

The medical model understands the embodiment of the disabled person primarily in terms of physical impairment caused by some underlying pathology. One of the many problems with such an understanding of disability is that it conceptualizes the body of the disabled person from the outside in terms of clinically observable impairments or loss of function relative to some normal or pre-existing state. Such a third-person understanding of the body of the disabled person fails to recognize their lived embodiment.

This understanding of the disabled person’s embodiment from a third-person, objectifying standpoint is shared by the social model. Thus the British-based disability movement, the Union for the Physically Impaired Against Segregation (UPIAS) defined impairment as “the lack of a limb or part thereof or a defect of limb, organ or mechanism of the body.” Disability is distinguished from impairment as “a form of disadvantage imposed on top of one’s impairment.” But as Hughes and Paterson (1997) noted this leaves in place a pathological and medical understanding of impairment. According to the social model, disabled people embody certain biological properties that are classified as physical impairments. The social model claims that based on such a classification disabled persons are then subjected to social forms of prejudice, exclusion and oppression. The limitations disabled people experience are thus traced to their social circumstances while the lived experience disabled people have of the world through their embodiment in it is at best sidelined and ignored^8^. But as Silvers (2010) has noted: “to ignore experiences of being weak, enervated, in pain and vulnerable in modeling disability is deceptive because these are the most salient experiences in most, or at least in many disabled people’s lives” (Silvers, 2010, p. 20). Everybody has such experiences from time to time. The worry Silvers is articulating is that such experiences may so permeate the lived experience of some disabled persons as to make their lived embodiment different in kind.

The problems we have just described stem from a medicalized understanding of embodiment in terms of physical impairment. Both the medical and the social model conceive of the disabled person’s physical impairment from a third-person, objectifying standpoint. What such an objectifying conception of the body misses is how the impaired body is also the medium of the disabled person’s experience of the world. In the next section we outline the Ecological-Enactive model of disability, which offers a different perspective on the embodiment of disabled persons, one that is better placed to do justice to how the body of a disabled person situates them in the world.

## The Embodiment of Disability

The bodily impairments that occur in disability are standardly understood as biomedical clinically observable pathological conditions that cause limitation in capacity or problems in performance (see Boorse, 2010)^9^. Of course there can be impairments or loss of ability that are non-medical but these do not lead to disability. A pianist for instance can lose their finger dexterity through lack of practice leading to an impairment in their ability to play the piano. It is typically assumed, however, that any bodily impairment associated with disability must be a clinically observable medical condition. An example of this type of reasoning is the attempt to specify the meaning of the terms ‘disability,’ ‘impairment’ and ‘handicap’ for health professionals by the World Health Organisation. They define the impairments relevant to classifying disabilities as “the loss or abnormality of psychological, physiological, or anatomical structure or function.” (*International Classification of Functioning, Disability and Health*, 1980, p. 27).

By understanding the embodiment of disabled persons in terms of impairment, both the medical and social models of disability pathologize the embodiment of disabled persons. The implicit contrast is with the non-disabled, “normally” embodied person whose psychological, physiological and anatomical structures and functions are intact and serving a proper function. The notion of bodily impairment assumes a distinction between normal and pathological embodiment. But how is this distinction to be understood? Is it really coextensive with the distinction between the disabled and non-disabled?

We’ve seen above how the normal body is typically understood statistically in terms of what is average relative to a reference group (Buchanan et al., 2000). There is assumed to be a standard of normal functioning for humans such as for example sightedness. Disabled people are taken to be impaired insofar as they are embodied in ways which depart from this standard. The blind for instance will count as disabled because they lack the capacity to see, while people that wear spectacles will not count as disabled.

We will follow Canguilhem (1991/2015) in reversing the relation between the normal and the average. Instead of defining the normal in terms of the average, Canguilhem proposed to understand the average in relation to what is normal (Canguilhem, 1991/2015, p. 156). A human trait such as average lifespan in a given population is not normal because it is frequent, but is frequent because it is normal (*Op cit*, p. 160). This is to say that it is normal relative to a form of life – the regular and relatively stable patterns of behavior found within a population. The average duration of a person’s life for instance varies in ways that depend on many different factors including the society to which the person belongs, their class, and occupation. These factors vary across and within populations depending for instance on:

“the techniques of collective hygiene which tend to prolong human life, or the habits of negligence which result in shortening it, depending on the value attached to life in a given society, are in the end a value judgment expressed in the abstract number which is the average human lifespan. The average life span is not the biologically normal, but in a sense, the socially normative, life span. Once more, the norm is not deduced from, but rather expressed in the average” (Canguilhem, 1991/2015, p. 161).

The statistical understanding of normality has it exactly backwards. The average is to be understood in relation to what is normal given social practices, such as practices of hygiene and medical practices, and not the other way around. Moreover, what is normal depends on the norms and values that people follow. Practices of hygiene such as washing one’s hands are normative in the sense of specifying what a person should do if she is to act in agreement with the values of her community^10^.

The distinctions between normality and pathology and health and illness are thus not to be understood in terms of a statistical deviation relative to some reference class. The embodiment of the disabled person should not be understood in terms of a generic bodily impairment. We should instead understand the distinction between normality and pathology in relation to an individual organism and its capacity to adapt to its environment, which in the case of humans is a sociomaterial environment. It is a defining characteristic of life that the organism can establish norms and values that arise from the organism’s need to maintain its dynamic stability with the environment. The organism has an interest in and cares about its own continued existence. The concern for its existence is an intrinsic value for the organism borne out of the need for the organism to continuously take action to maintain its integrity. As Canguilhem noticed: “life is not indifferent to the conditions in which it is possible” (Canguilhem, 1991/2015, p. 126)^11^. If a living body is to remain viable over time, the organism must regulate how it engages with the environment based on changes in its internal or external conditions. The organism can sense that it is hungry, thirsty, sick or fatigued, for instance, or it can sense joy, pride, frustration or satisfaction in its actions. In short, the organism has an evaluative perspective relative to which it evaluates how it is faring in relation to its environment. At minimum, an organism should regulate how it acts so as to bring about an improvement in its circumstances, while at the same time avoiding threats to its own continued viability.

We will refer to the organism’s capacity to distinguish between situations as for example improving or deteriorating as “bodily normativity.” We describe the organism’s evaluative capacity as “normative” because it is a guide to how the organism should act if is to meet the demands of its environment and remain in harmony with it. Bodily normativity is what enables the organism and the environment to continuously form a temporarily stable equilibrium with each other^12^.

Crucially, humans don’t only care about their own continued existence. They care about a wide variety of activities because over the course of their lives they develop skills for acting well in many different contexts and situations. We should not think of bodily normativity as a capacity belonging to individual persons distinct from social and cultural normativity – the rules the person “follows” (Wittgenstein, 1953) as she engages in social life. People grow into and become skilled participants in a multiplicity of different practices in their everyday life. By taking part in communal customs and practices the person develops what we can describe as a “situation-specific discernment,” a feel for how they should act in a particular situation (Rietveld, 2010). Bodily normativity in humans takes the form of an ability for distinguishing, typically in the flow of activity, between better and worse, appropriate and inappropriate, what is significant and worth paying attention to and what is not. Humans by taking part in many different activities develop a multiplicity of different (and sometimes conflicting) cares and concerns that feed into their sensitivity to how they are faring in life. People are normally able to act adequately in a given situation because they embody a concern for what counts as adequate action within a practice. Given that the practices humans take part in are holistically related – they form what Wittgenstein (1967, p. 108) described as “the whole hurly burly of human activity” – these multiple cares and concerns must be integrated in a person to form a “single complex sensitivity” (Rietveld, 2010)^13^.

Based on bodily normativity, multiple affordances the environment offers will stand out for an individual as calling for action. We borrow the notion of “affordance” from ecological psychology to refer to the possibilities for action the environment makes available to an animal belonging to a form of life (Gibson, 1979; Rietveld and Kiverstein, 2014).^14^ The multiple affordances that invite the individual to act we describe as forming a field of relevant affordances (Rietveld et al., 2018). The individual’s discernment for what it ought to do in a particular situation can thus be understood in terms of its skill-based, selective responsiveness to a field of relevant affordances. Based on their sense of what a practice requires of them and the care they cultivate by taking part in practice certain affordances will stand out as inviting action. Insofar as they embody a single complex sensitivity, they will be ready for engaging with a holistically structured field of multiple relevant affordances.

Canguilhem described the person in good health as feeling “more than normal – that is, adapted to the environment and its demands - but normative, capable of following new norms of life” (Canguilhem, 1991/2015, p. 200). He gives as an example an organism that is forced to resettle at a higher altitude after being accustomed to living at the sea level, perhaps because of the flooding of its natural habitat. The variation in its environment implies a change in oxygen concentration in the air, different food, ambient temperature, *etc*. In order to flourish in these new conditions, a healthy organism would institute new norms to compensate for the changes in its habitat. It will need to select from among the new possibilities, what is good and exclude what is bad for it. If the organism is incapable of producing new norms to adapt to the new environment, that organism would go from a normal state in the previous environment to a pathological state in the new one. The healthy organism, the one that, in this case, can move from a lower to a higher place. It is ‘more than normal’ in the sense of being able to adapt to a variety of ensuing events. What the organism does can be described as “more than normal” because it is able to find new ways of doing things that are better suited to this environment than their previous habitual ways of doing things.

We suggest Canguilhem’s idea of *health as the experience of being more than normal* is an important clue for how to understand the embodiment of disabled people in ecological-enactive terms. Such an account will improve on the medical and social models understanding of embodiment in terms of bodily impairment because it will allow us to make a distinction between normal and pathological embodiment.

## Normal and Pathological Embodiment: Toward an Ecological-Enactive Model of Disability

To be normal is for a living body to be able to maintain a state of dynamic stability with its environment. This is something the organism needs to continually reestablish by regulating its engagement with the environment based on bodily normativity. The stability the organism achieves is always hard won under conditions of continuous change, which is why we describe it as a “dynamic” stability. The organism must therefore always be ready to act not only in familiar circumstances it has encountered regularly in the past, but also to adapt its activities to novel situations that differ from anything the organism has hitherto encountered. Indeed human agents in adapting their activities to the particularities of a given situation cannot just repeat what they have done in the past. They must adapt what they have done in the past to the particularities of the situation that now confronts them. Think about bicycling through a busy city. You follow the same route but under unique traffic conditions that will never be repeated in exactly this form again. To maintain a state of equilibrium the agent must adapt what they have done previously to these unique and often unrepeatable conditions. To respond adequately to affordances as they take shape in this particular situation will often require the organism to risk, and be tolerant of potential failure. You may for instance almost collide with another cyclist talking on their mobile phone, or with a pedestrian that casually walks into the cycle path. Yet most of the time you succeed in avoiding injury by spontaneously taking measures that allow you to skillfully correct for such incidents as they arise.

It is this capacity of the living body to continuously restore dynamic stability by adapting in better or worse ways to uniquely occurring conditions that we take to be a defining characteristic of a healthy living body as contrasted with one that is sick. Canguilhem in the passage we quoted from above associates health with the capacity to follow “new norms of life” (*Op cit.*) – a capacity he associates with what we have called *bodily normativity*. Being healthy means being able to establish a state of dynamic stability with the environment, and not only in the present situation, but also in a near open-ended range of other situations going into the future. The bodily normativity that governs the organism’s engagement with its environment will need to be made anew on each occasion because dynamic stability is continuously achieved anew, often under uniquely occurring and unrepeatable conditions. The norms that regulated an organism’s conduct in the past are a guide to what the organism does in the present, and going into the future. They cannot however fully determine what the organism does in other possible situations the individual might find themselves in. To achieve dynamic stability in these other possible situations will call for transcending the norms that have governed the organism’s activities in the past, and the following of new norms that allow the organism to establish dynamic stability under the new and often unique and particular circumstances it now encounters. To adapt to changing conditions, the organism must be ‘more than normal,’ that is, capable of adapting not only to the demands of its current situation based on what it has done in the past. It must be “normative” in the sense of being able to institute new norms that allow it to reach dynamic stability in a changing environment in which it is confronted with novel situations.

This ability to adapt to change is transformed in illness into a capacity to limit or avoid change. The organism’s relationship with the environment is qualitatively different from that which it can accomplish when in a healthy condition. Canguilhem suggests that a person is sick when they can no longer exercise the capacity to follow new norms of life (Canguilhem, 1991/2015, p. 186). Instead, the person organizes their life around a single norm – the avoidance of situations they might generate what Goldstein referred to as “catastrophic reactions.” Goldstein distinguished “ordered” from what he called “disordered” or “catastrophic” reactions. Ordered behaviors are experienced “with a feeling of smooth functioning, unconstraint, well-being, adjustment to the world, and satisfaction” (Goldstein, 1934/1995, pp. 48–49). In catastrophic reactions the person feels “unfree, buffeted and vacillating” because it is unable to respond adequately to situations it could have ordinarily dealt with when healthy (*Op cit*, p. 49; Goldstein, 1940, ch. 4). The living body is unable to establish a dynamically stable relation with the environment in situations in which this would normally prove possible. As a consequence the person experiences the environment as dangerous, a threat to their existence. Consider Goldstein’s description of the reaction of one of his patients with a lesion in the cerebral cortex when he tries to perform an easy arithmetic task and fails.

“By simply looking at him we discover a great deal more than his arithmetical failure. He looks dazed, changes color, becomes agitated and anxious, starts to fumble. A moment before, he was amiable; now he is sullen and evasive or exhibits temper. He presents a picture of a very much distressed, frightened person, a person in a state of anxiety. (…). We may call the state of the patient in the situation of success *ordered behavior*; his state in the situation of failure, *disordered* or *catastrophic behavior.*” (Goldstein, 1940, pp. 85–86).

Anxiety may arise in very innocent circumstances for a person in a pathological state. If the person anticipates being unable to adequately respond to a situation (e.g., solve a simple arithmetic problem), the situation becomes very threatening for him, and anxiety will block his capacity to perform at all (Of course it can also be the patient has panic attacks every now and then and in the meantime can function quite normally). The avoidance of such challenges becomes the sick person’s way of being-in-the-world. They live a life in which they keep everything in the environment as stable as possible, and avoid at all costs unfamiliar things and events. Goldstein reports how patients of his would avoid taking walks because simply going for a stroll could lead to unexpected encounters, and catastrophic reactions. Even unfamiliar routes around the hospital were avoided (Goldstein, 1940, p. 100). Living according to the norm of avoiding change amounts to a shrinkage of the possibilities for action the individual is open to acting on. A pathological living body achieves a state of dynamic stability only by arranging their affairs so as to keep the environment as constant as possible at the cost of explorative engagement with the world that would normally also lead to new skills. The person acts more generally with the aim of keeping themselves in situations they can adequately manage given their illness. Illness is characterized by a stagnation of life in which the person restricts their engagement with the environment with the aim of avoiding catastrophic reactions.

Our Ecological-Enactive model uses Canguilhem’s analysis of health and illness to distinguish “normal” from “pathological” modes of living embodiment. A person is normally embodied if they can adapt their activities adequately not only in response to the particularities of their current situation, but also in responding to a near open-ended range of alternative possible situations. They are able to transcend their current situation as is shown by their readiness to respond adequately to many other eventualities and possibilities. They can, in Canguilhem’s terms, “institute new norms in new situations” (Canguilhem, 1991/2015, p. 197) By contrast, when a person is unable to institute and follow new norms and instead acts exclusively on the basis of the norm of avoiding adapting to change then we will describe them as being pathologically embodied. A person is pathologically embodied when they morbidly avoid situations that could lead to catastrophic reactions by withdrawing from life, confining themselves to regimented and ordered situations which they can manage^15^.

We’ll argue in the next section that disability (at least in people with CP) doesn’t necessarily entail pathological embodiment because impairment doesn’t necessarily lead to a shrinkage in the environment, and the withdrawal from life characteristic of pathological embodiment. We make this argument by contrasting reports from subjects with CP. One of the individuals reports experiences of pathological embodiment. The other individuals whom we interviewed using the phenomenological method described in the introduction, we will suggest, are best interpreted as describing experiences that correspond to normal embodiment.

## Pathological and Normal Embodiment in Persons With Cerebral Palsy

We begin this section by considering the case of Michael as an example of pathological embodiment in Cerebral Palsy^16^. Michael’s CP has led to left-sided hemiplegia, which highly limits what he can do with his left hand. He also needs a walking stick to walk. This is how he describes his relation with his environment:

“The world, that is, my surrounding environment, appears as something hostile, which I am a part of, but certainly not ‘in.’ The world is an object I continually manipulate, rather than being a friendly place and somewhere I feel at ease or even at home. Within this hostile world, other people appear as obstacles to be avoided, not just because I fear bumping into them and hurting myself, and them. Even a hand offering help with shopping bags can appear hostile as it is an unexpected disruption to my ‘walking plan’. I live in a world which assails the body and self, and I can only hope that the adjustments will allow me to survive” (Cole et al., 2017, p. 2).

The hostile world that Michael describes mirrors Canguilhem and Goldstein’s description of illness. Recall how the sick person tries at all costs to keep the environment as stable and predictable as possible in order to cope with life. Michael’s embodiment is pathological when he is unable to adapt what he does to unexpected change. He doesn’t feel capable of coming up with different and better ways of doing things. He lives his life in a “surviving mode,” and in that mode of being, the environment is polarized in a way in which all that matters is for him to keep himself alive. This existential feeling of being constantly under threat (Ratcliffe, 2008), and on the verge of anxiety and catastrophic behavior is clearly described by Cole et al. (2017) in relation to Michael:

“What cannot be over-emphasized is the existential nature of inhibited intentionality. The difficulties the walker [Michael] faces threatens not only his agency, his ability to commune with other human beings, but also his very existence. Walking down the street is about way more than just walking. Inhibited intentionality shrinks one’s social world” (Cole et al., 2017, p. 3).

Michael’s lived experience contrasts markedly with the descriptions of a number of persons living with CP we interviewed. The experiences they described make it clear that they are able to skillfully explore for new affordances in order to establish new and better ways of doing things. This is the essence of normal embodiment as we have described it above. Thus consider *SG*, a 28-year-old woman with spastic cerebral palsy that affects motility in her legs and her right arm. The following is the description *SG* gave in the context of a phenomenological interview of her experience of shaking hands and how it has evolved through time, how she has learned to deal with the challenges of meeting a new person, or greeting someone at a party:

“I’d definitely rather just shake hands with my left. I don’t know if you noticed, but I shake hands with my left, because that’s the side I prefer to show of myself. I’m more confident in the meeting when I know people, if I’m allowed to give my left hand rather than my right. But it’s taken me so many years to figure out that I can just give the left hand, because I’ve always given the right one, and it’s always been like “ugh!” meeting new people. At a huge birthday party where you don’t know anyone and have to go say “Hi, S” while keeping balance on my rollator as well. So in reality it’s a question of balance. Then I’ll use the left so I can stabilize with the right. It was a huge relief for me to find out I could just give the left hand! There wasn’t much to it, because it just feels more comfortable for me, and that’s really what matters.”

Notice SG’s attitude toward her impairment. She realizes that shaking hands with her right hand – a social convention in the western world – implies that she can’t easily maintain her balance. Thus, her right hand will not be the best hand to offer to the person she is greeting. Shaking hands with her right hand feels wrong. She feels much better as a whole – she can maintain a better bodily equilibrium, look at the other person’s face, and so on – when she offers her left hand. This is something she found out after a long time of shaking hands using her right hand. It happens every now and then that one encounters a person who for some reason (perhaps an injury or because they have their hands full) shakes hands with their left hand. Thus, SG did not need to come up with a completely new pattern of social engagement. She just needed to be open to a non-standard, unconventional way of doing things, others likewise have recourse to on occasion.

SG describes a similar experience in the context of the experiment we described in the introduction. The task she refers to consists of lifting a tray on which is placed a cup of water. Initially she struggled to perform this action. Here she describes a feeling of discontent she experienced when performing the action with her father:

“...*I wanted to try and see what would happen if I only did it with my left. And I could feel it was more insecure, because the glass with water created some balance issues.*”

Afterward, she performed the same task with the therapist. Here she reports her experience of having found a manner of performing the action that worked better for her:

“*Suddenly it dawned on me that I had done it this way every time, and now I could do it differently. I hadn’t even thought that you could do it that way! So, in the middle I stopped, because I had time to think “God, you’re right,” but I had already begun the action! And really it was because it dawned on me that I could do it in a different manner than I thought!*”

Despite the movement limitations SG experiences, she’s still able to be spontaneous in the performance of the tasks at hand. She is constantly looking for better ways to perform the exercise, and even though she might find a way in which she feels comfortable performing, she feels she can keep looking for better ways to perform an activity. Sometimes she fails to improve, sometimes she succeeds in coordinating her actions to new affordances that allow her to establish a new way of acting. This flexibility and adaptability to upcoming challenges is absent in what we have described as pathological lived embodiment, as seen in the case of Michael above. Every unforeseen event – even a helping hand – can become an insurmountable challenge that can trigger severe anxiety the person acts to avoid. Instead of being open to exploring for affordances that allow for the formation of a temporary stable equilibrium with the environment, the person acts to limit to the best of their ability, situations in which they are unable to respond adequately^17^.

For a person with CP, performing tasks with the impaired limb will typically prove to be suboptimal, compared with how they would perform them with their unaffected limb. They can however often face the task with some degree of openness to what might happen, without panicking. They are open to finding ways to perform the task that work better for them. This often calls for creative improvisation if they are to avoid getting into trouble. Yet they are prepared to take risks in spontaneously adapting to the demands of the situation, modes of engaging with the environment characteristic of healthy or normal embodiment. Recall *CN* – the woman we quoted from earlier describing her experience of horse-riding. This is how she described her experience of the tray exercise of the tray exercise when she does it with her mother:

“*I think that we just do it, because we know each other so well. We just do it. We know where the limits are, and what you do in those cases. So I don’t think there’s any challenge in it. Of course, when you change with your left hand and such, and optimally I’d grab the tray with my right hand independently of which way it was going. If you hadn’t said that I should grab it with my left, I would have grabbed it with my right. So, I don’t think there’s anything challenging or uncomfortable in this.*”

Based on the experiences we have described in this section, we can identify two features distinguishing normal from pathological forms of embodiment in these persons. Normal embodiment occurs in people with CP with a preserved capacity for adapting their manner of engaging with the affordances of the environment so as to find the affordances that work for them. Second, the normally embodied person should be ready to test established patterns of activity to the best of their ability when circumstances call for them to do so. They should be able to explore for better ways of engaging with the relevant affordance that correct for discontentment with their previously established ways of doing things. These features of normal embodiment and normal experiences have been deeply investigated both in phenomenology in relation to the lived body, and in Ecological-Enactive cognitive science in relation to the living body. In the next section we will make use of these accounts to round off our argument that people with CP can be normally embodied despite their physical impairments.

## Normality as Optimality and the Tendency Toward an Optimal Grip

Following Canguilhem we’ve suggested that a key feature of health from an Ecological-Enactive perspective is to transcend what in the current situation is experienced as normal in readiness for a near open-ended number of other possibilities that may lie on the horizon. We experience a situation in a manner that deviates from what is optimal. The person is then drawn into action by relevant affordances in such a way as to temporarily restore dynamic stability. Merleau-Ponty gives the example of standing too close to a painting you are viewing in a gallery (Merleau-Ponty, 2012, p. 315). You experience a tension in relation to the painting, and you step back so you can better see the details in the context of the painting as a whole. Merleau-Ponty understands life as a process always delicately balanced between relatively stable equilibrium with the environment, and disequilibrium, or instability (Merleau-Ponty, 2003, p. 149; Rietveld, 2008b; Kiverstein and Rietveld, 2018). Organisms compensate for this inherent disequilibrium through movement. When faced with the tension generated by disequilibrium, the norm will be for the organism to act in order to relieve the tension so as to move in the direction of “the optimal conditions of its activity” (Merleau-Ponty, 2012, *Op cit.*). Relevant affordances stand out for the agent from their surroundings based on divergence from an optimal condition. The organism is continuously being moved to get ready for action possibilities that can contribute to reducing divergence from a state of relative equilibrium. They will normally tend toward an optimal grip on a whole field of relevant affordances (Rietveld et al., 2018).

The normal lived-body is the one that *tends toward* optimality, creating new and better bodily norms to guide its activities. Steinbock (1995) in discussing Husserl’s account of perceptual normativity has described this dynamic well when he writes:

“From one perspective experiences are ordered according to the previous norm; from another, they actually surpass it such that the old order refers to the new as norm; the former as abnormal, the newer as normal.” (Steinbock, 1995, pp.146–147).

To make the same point in our Ecological-Enactive terms, the skilled individual is able to adapt to an environment in flux by sometimes exploring for unconventional possibilities. They will creatively establish novel ways of engaging with the environment by expanding their set of skills (including fine-tuning an existing skill).^18^ For disabled people, it is very important to explore for new and improved ways of doing things by trying out what is possible, as well as having a practical knowledge of her own bodily capabilities, skills, and limitations. Consider in this light the following remark of CN reporting on her experience of passing and receiving a cup of water in our experiment:

“*I think more about how to grab it without everything going wrong. I knew that I would never grab around it, because then there’ll be water everywhere and I’ll be wearing wet pants for the rest of the day. (…) So of course you think about how to solve it. And you also do that day to day.*”

She did not stick to her pre-established routine ways of engaging with the world, but was open to exploring for new ways of doing things that work better for her given the constraints of her physical impairments. We think it makes sense to describe her as tending toward an optimal grip on the possibilities that matter to her, correcting for disequilibria as they arose in her engagement with the world. The normal is not only the optimal as it is found in the person’s present circumstances, but also the capacity to transcend what counts as optimal in the present situation. The person must also be open to engaging with previously unexplored affordances as they are encountered in the future. A disabled person, we will argue, can be considered normally embodied just so long as she is able to adequately adapt her actions to the particular situation in which she is acting. This may call for her to break with how she has done things in the past and open herself to new affordances that allow her to improve her skills. Then, even if the disabled person is less flexible compared to the non-disabled person, she is still able to tend toward an optimal grip in adapting to the demands of her environment.

Thus, returning to our Ecological-Enactive account of normal embodiment we gave at the end of the previous section, we can redescribe the two dimensions of normal lived embodiment as follows. Crucially, these dimensions are also manifest in the experience of people with CP we interviewed:

(1) The agent is able to tend toward what is optimal in their lived experience of the world. They are capable of adequately engaging with multiple relevant affordances in the practical contexts in which they are to be found.
(2) They can transcend what is currently optimal in their active engagement with the world by exploring responsiveness to unorthodox affordances and/or developing/enriching abilities to *establish new and ‘improved’ possibilities for engagement* going into the future.

Normal embodiment doesn’t mean lack of difficulty in performing daily tasks. We continuously encounter obstacles and have to correct for action slips and failures in normal everyday life. It is usual for a person with CP to find daily activities more challenging than a non-disabled person. But, even when faced with very difficult activities, it is inherent to normal embodiment to be able to explore in search of affordances that allow the individual to tend toward an optimal grip. It is important to consider that CP is a congenital disorder, which leads the person with CP to develop from the very beginning an open attitude to risk-taking, often exploring alternative strategies to deal with daily challenges (see Martiny, 2015). A good example of this is *KR*, another participant in our experiment. *KR* is a middle-aged man with CP with dystonia in his left arm, which limits to a high degree his arm and hand movements. Faced with the task of passing and receiving a cup of water in our experiment, he struggled considerably. This is how he described the experience after having performed the task with his impaired hand by just pushing it, instead of grabbing it.

“*It’s impossible if I had to take it. It would be very demanding. Anything is possible, but*… *I think it may be possible that I take the one with water in and lift it, but I would have to carry the left arm, put the hand down the cup and push on the thumb, and I would probably still spill half the water.*”

And he adds later in the interview:

“*The water part was pretty much an impossible task. (…) You have to be creative. Push it. I’ll have to do it that way, then. There’s a solution to all problems.*”

*KR* exemplifies the experience of facing a demanding task while holding on to the conviction of being capable^19^. It is a matter of trying different actions, to find a solution, and also to keep trying to transcend the currently established ways of tending toward an optimal grip. In the first round of tasks, KR didn’t spill water in the passing and receiving of the cup, mainly because he did most of the work with his other hand, while barely grabbing the cup with his impaired hand. In the second round, he tried to do the same task mostly with his impaired hand, knowing that it was a riskier strategy:

“*When I had to give the cup back, I did it differently. It was almost conscious, because I wanted to do it differently than I had done it last time. (…) I thought that the last time I didn’t spill, so now I wanted to see what would happen if I did.*”

We’ve shown how people with CP can overcome the challenges they are faced with in daily life so as to exemplify the two dimensions of normal embodiment we’ve identified. Thus, although dealing with daily life undoubtedly brings with it many challenges, the person with CP need not be thought of as being pathologically embodied. They do not necessarily experience the anxiety that comes with the failure to adapt to change, but on the contrary are often ready to risk failure in exploring in search of affordances that allow them to tend toward an optimal grip. With the help of the phenomenological tools and the EE account of normal embodiment we’ve proposed, we have shown how it makes sense to think of many people with CP as normally embodied.

## How to Understand the “Dis” in “Disability”?

There is an apparent tension between the notions of disability and normal embodiment we would like to end by discussing. If, as we have argued, a person with a physical disability like CP can still be considered normally embodied, why do we describe these persons as disabled? We aim in this section to understand disability from a first person perspective. From a phenomenological point of view, we suggest that disability is a form of self-experience. Being disabled can be described in terms of the experience of I-cannot.^20^ The experience of ‘I-cannot’ permeates to different degrees the person’s practical engagements with affordances in the environment. Thus a person that experiences a speech impediment will for instance have an experience of I-cannot because of the norms of embodied communication, which are intolerant, and even hostile toward people that speak with a stutter. Here we are in agreement with the social model that the limitations disabled people live through are often “a consequence of the profound oppressions of everyday life” (Paterson and Hughes, 1999, p. 603).

We in no way mean to deny or downplay the difficulties and challenges people with CP face and often overcome. Physical disabilities more generally, are directly linked with activity limitations, and suffering that is not experienced by non-disabled people. The ‘dis’ in disability is to be taken seriously. Nevertheless, disability doesn’t necessarily lead to an inability to tend toward an optimal grip on a field of relevant affordances. It is still possible for many people with CP to be open to exploring the affordances for the environment they care about, and to engage with them in different ways, and in ways that are adequate to the situations in which they are found. A key part of what disability means for a normally embodied person is, we suggest, constantly correcting for this experience of I-cannot. When they tend toward an optimal grip by finding their way to affordances that allow them to temporarily form a dynamic stability with the environment, they are overcoming the experience of I-cannot.^21^ Each time they find affordances that allow them to conquer instability, they are at the same conquering their experience of I-cannot. Thus an experience of I-cannot is quite consistent with a disabled person at the same time experiencing a world in terms of its affordances because of the skills they have developed.

The being-in-the-world of non-pathologically disabled people is, however, fundamentally different from that of non-disabled people insofar as the former must constantly conquer and reconquer the experience of I-cannot. A person with CP will always experience some challenges dominated by the feeling of I-cannot. By remaining open to different affordances, they are able to compensate for this feeling without experiencing a catastrophic reaction - the experience of being unable to adequately adapt to a situation. The affordances they make use of may differ from those exploited by people that are not disabled. We suggest that the I-cannot experienced by the non-pathologically disabled person can be understood as a local I-cannot, with a background of I-can: I-can do it in a different way, I-can ask for help, I-can do it slowly, *etc*. This contrasts with the experience of I-cannot of the pathologically embodied person, which deeply pervades her being-in-the-world. She lives in a dangerous and threatening world, and must structure her environment so as to avoid catastrophic reactions in which she is unable to establish a dynamically stable relation with the environment. It is this pervasive feeling of I-cannot that drives the pathologically disabled person to keep everything around her as stable as possible, in order to preserve the small and fragile region of I-can, and avoid life-threatening anxiety. This pathological preservation of the local I-can inhibits the person to transcend her way of engaging with the world in favor of better ways, thus preventing her from tending toward an optimal grip^22^.

## Conclusion

We’ve argued that disability doesn’t necessarily entail pathological embodiment based on the experience of people living with CP. We’ve done so by providing an Ecological-Enactive account of the person’s embodiment that allows us to distinguish between pathological, and normal forms of embodiment, while at the same time doing justice to the lived experience of disability. We’ve argued that people with CP are often normally embodied because they can find ways to tend toward an optimal grip on a field of relevant affordances. They can transcend the way they have done things in the past in order to explore for new affordances that allow them to adapt adequately to their situation. There are many factors involved in the capacity (or incapacity) of a disabled person to explore for alternative ways of dealing with daily challenges: not only the actual physical capabilities of the person, but also psychological, social and environmental factors that can encourage or discourage them to make the effort and tend toward an optimal grip. One serious concern with the medicalizing and pathologizing of disability is that it can turn a normally embodied disabled person into a pathologically embodied one by obstructing the person’s capacity to tend toward an optimal grip. The person can experience their impairment in ways that inhibit them from looking for alternative or unorthodox ways of engaging with the affordances in the environment and from developing new skills and abilities. Thus, consider SG’s description of the difficulties she experiences in passing a cup of water using a manner of grasping the cup her former physiotherapist describes as proper:

“*I got annoyed. My old physiotherapist would say “you can’t hold it like that! You need to do a proper grip.” And then I correct myself, because I’ve always been taught that I can’t do it like that, so of course I have to be able to do the other thing. And it’s a bad and a wrong way to do it. So really I correct myself in these situations.*”

If people with CP are not allowed to find alternative ways of dealing with daily challenges, if therapy hinders their capacity to explore and develop their own abilities that work for them given their embodiment, or if the sociomaterial environment is built around only able-bodied people, their practical engagement with the world will become much harder and the risk of becoming pathologically embodied will increase. This is because the way the disabled person conquers the experience of I-cannot is by finding their way to affordances that allow them to act adequately within the constraints of their impairment. The “proper grip” that SG describes her former physiotherapist as enforcing, is a socially accepted way of engaging with specific affordances in practical contexts. This proper grip, however, does not necessarily work well for the person with CP. If they are to succeed in tending toward an optimal grip this will often call for them to break with the established ways of doing things in their life-world. People with CP can adapt and develop new skills, but interpersonal relationships can still be challenging: It can be difficult for them to interact and coordinate with non-disabled people. This is mostly due to the fact that non-disabled people are not skilled in interacting with people with CP, and they bring with them a pre-established normativity that often conflicts with the abilities and skills developed by a disabled person to perform activities in everyday life.

Experiences of I-can and I-cannot are complex phenomena in which the person’s embodiment and skills are faced with the demands of an environment structured by sociomaterial practices. Pathological embodiment can arise out of sociomaterial practices that make it too hard or impossible for the disabled person to explore, and establish her own skilled ways of engaging with the relevant affordances, including the social affordances that materialize in interaction with other people. In a similar manner, a friendly, supportive and flexible sociomaterial environment can prevent a disabled person from becoming pathologically embodied. We’ve argued the lived embodiment of a person with CP doesn’t necessarily entail pathological embodiment. On the contrary, people with CP can still explore their environments for affordances that make it possible for them to live a rich and fulfilling life.

## Data Availability Statement

The datasets generated for this study are available on request to the corresponding author.

## Ethics Statement

Ethical review and approval was not required for the study on human participants in accordance with the local legislation and institutional requirements. The patients/participants provided their written informed consent to participate in this study.

## Author Contributions

JT gathered and analyzed the experiential reports of people with cerebral palsy. JT and JK co-authored the manuscript. ER provided feedback and helped to refine the argument of the manuscript.

## Conflict of Interest

The authors declare that the research was conducted in the absence of any commercial or financial relationships that could be construed as a potential conflict of interest.

## Footnotes

1 We follow Barnes (2012) in using the term ‘disabled people’ rather than ‘people with disabilities’. She writes: “I use ‘disabled people’ rather than ‘people with disabilities’ because ‘disabled people’ mirrors our usage of other terms which pick our minority social groups – for example, we say ‘gay people’ not ‘people with gayness’. It is sometimes suggested that we should say ‘people with disabilities’ because ‘disabled people’ suggests that disability somehow defines the person. But I simply don’t think that’s true. Saying that someone is a disabled person doesn’t mean that disability defines who they are anymore than saying that someone is a gay person means that sexuality defines who they are.” The term ‘people with disabilities’ is often associated with the medical model. However, it should be noted this is not the case in Denmark for instance. There the disability movement has fought for the term ‘people with disabilities’, to avoid being objectified as ‘disabled’, and to call attention to the fact that the person’s identity goes beyond disability. Our thanks to Jacob Nossell and Kristian Martiny for discussion of this issue.

2 We assume here that enactive cognitive science and ecological psychology can be smoothly integrated (see Kiverstein and Rietveld, 2018 for an argument for this assumption). It should be noted, however, that such an integrative project is not without its conceptual tensions. In their ground-breaking work *The Embodied Mind* Varela et al. (1991) expressed doubts about the realism of Gibson’s ecological psychology. They argued that Gibson’s ecological theory of perception was premised on a one-sided view of the animal-environment relation. Varela et al. (1991) argued instead for a view of the animal-environment relation in terms of a structural coupling and codetermination. Ecological psychologists for their part have objected that the enactive notion of sense-making implies a view of the physicochemical environment as meaningless until it is given meaning by the agent. Enactive cognitive science from their perspective fails to do justice to the pragmatically structured environment, and the role of the environment in constraining and enabling behavior. These traditional tensions notwithstanding we pursue the possibility of a collaborative and integrative research program in this manuscript. The task of smoothing these conceptual tensions will have to await another occasion [but again see Kiverstein and Rietveld (2018) for first steps in this direction]. We focus here instead on putting such a program to work to show how to avoid pathologizing the experience of being disabled, an urgent ethical and political matter of concern.

3 This experiment was designed and performed by an interdisciplinary team of researchers affiliated with the University of Copenhagen – Center for Subjectivity Research, the Technical University of Denmark, the Elsass Institute, and the Enactlab.

4 All measurement procedures contributing to this work comply with the ethical standards of the relevant Danish committees on human experimentation and with the Helsinki Declaration of 1975, as revised in 2008. Ethics approval is not obtained for this experiment because, according to the Danish ethics committee law §2, no. 1, only health scientific research that includes biological material and clinical trials needs approval, whereas qualitative interviews and non-health scientific measurements (eye-tracking and motion capture) do not need approval. The treatment of data complied with the General Data Protection Regulation. The participants filled out a form providing informed consent, and the Elsass Institute, where the experiment took place, approved of it.

5 We explain in detail the conceptual framework and the methodology of the experiment in Martiny et al., Unpublished.

6 For a detailed account of the methodology and the theory behind the phenomenological interviews, see Høffding and Martiny (2016). See also de Haan et al. (2013, 2015) for an application of phenomenological interviews with OCD patients treated with Deep Brain Stimulation.

7 Work in the philosophy of psychiatry has shown that to do justice to the complexity of mental illness will require eschewing a simple reduction of mental illness to biological dysfunction, and similar arguments could be made for disability. We thank a reviewer for this suggestion. Murphy (2006) shows for instance how a scientific psychiatry will have to make reference to many different physical, psychological, and social factors. The explanation of the causal etiology of mental illness will have to advert to many different variables – biological, psychological, and social – to account for illness symptoms. What is distinctive about a medical approach to mental illness according to Murphy is that it aims for a causal understanding of mental illness through constructing a causal model of the illness. The medical models of mental illness and disability share a view of people with mental illness as being “worse-off” in some way as a consequence of their bodily impairment. They are disadvantaged relative to the non-disabled population as a consequence of “the failure of their physiology or psychology to perform a natural function” (Murphy, 2020). The dispute between the medical and social models is thus not about the role of social and cultural factors in causing disability. It is instead about whether disabled people are intrinsically worse off because of their bodily and functional impairments. The social model denies this and claims instead that disabled people are worse off because of how society treats them (see e.g., Barnes, 2016).

8 To respond to this line of criticism, many theorists influenced by the social model have developed accounts that include the disabled person’s body understood in terms of its impairment. In doing so, some of them have taken distance (to different degrees) from the social model, aligning themselves with critical disability studies (Shildrick, 2009) feminist philosophy of disability (Tremain, 2017, 2019), crip theory (McRuer, 2006), social constructionism (Barnes, 2016) among other movements, with evident overlap between them. Tremain, for instance, develops a feminist account of disability based on Foucault’s notion of apparatus, which, she claims, is not vulnerable to the objection leveled against the social model that it “denies impairment and the body” (Tremain, 2019, p. 144). Tremain (2019) describes how the body and its material experiences cannot be dissociated from discourses, institutions, architectural forms, laws, scientific practices that bring this body into being as an impaired body. Notice, however, that the material experiences of the body she aims to accommodate are the experiences of a body brought into being by historically contingent practices as a kind of thing – an impaired, gendered, sexed thing (Tremain, 2019, *Ibid*). She rightly calls into question essentialist understandings of impairment but her critique targets an understanding of the body of disabled persons as an impaired thing. In a related vein, the crip theory, that inscribes disability within the queer movement, focuses on how neoliberal capitalism has been the force against which disabled and LGBT people have defined their identities. In this way, crip theory is mostly concerned with the institutions that enforce able-bodiedness and heterosexuality, like educational, religious and financial institutions, among others (see McRuer, 2006). We in no way wish to detract from these politically important arguments. However, when it comes to understanding the embodiment of disabled persons we align ourselves with theorists influenced by the social model that have considered crucial in their accounts the lived experience of disabled people (see Thomas, 2007; Shildrick, 2009; Silvers, 2010; Reeve, 2012).

9 Boorse notes that “the standard use of ‘impairment’ for biomedical aspects of disability is curious, for there is little reason to think it is a biomedical term at all, let alone a crucial one…I do not know how ‘impairment’ got its current role as general biomedical term in the disability literature. No doubt one motivation was that many disabilities – paralysis, blindness, missing limbs – are associated with static defects, not disease processes.” (Boorse, 2010: p.61). Boorse objects that the biomedical understanding of impairment as a clinically observable pathological condition is too broad – a “gross-dysfunction test may well give most people a disability.” (Boorse, 2010, p. 66)

10 Conceiving normality as we propose here is consistent with the idea that rigid or very conservative societies sometimes pathologize behaviors that deviate from what is considered normal (see Foucault, 1991). Non-conformists are sometimes blocked by other members of a practice from exercising their capacity to establish new norms. Think about how homosexuality was classified as a mental disorder until 1973. Some ‘deviations’ will, however, gradually be integrated and assimilated by a society and come to be considered normal. How fast this happens will depend on the political, economic and religious forces at play. The development of the queer movement is a good example of how societies progressively acknowledge and integrate behaviors that were once considered deviant and pathological. For an analysis of the sociological relations between homosexuality and disability, and how both have been pathologized, see McRuer (2006).

11 Canguilhem is anticipating here key ideas in ecological-enactive cognitive science [c.f. Merleau-Ponty (1942/1963) on the difference between the physical and living orders]. In our work we’ve shown how norms and values are intrinsic to the organic living body that has a sense of how well or badly things are going in its engagement with its environment. This is the evaluative dimension that is intrinsic to their engagement with the environment. The individual may sense a disequilibrium that tells them all is not quite right. The norms in play in the individual’s engagement with the environment are situated: they concern what the individual should do to improve its situation to correct for a sensed disequilibrium (Rietveld, 2008a; Rietveld and Kiverstein, 2014; Bruineberg and Rietveld, 2014). The norm here relates to the adequacy of the individual’s grip on a situation. The individual’s grip is adequate, or inadequate, better or worse. There is an important connection between our idea of situated normativity and what enactivists call adaptivity (Di Paolo, 2005; Thompson, 2007; Di Paolo et al., 2017). Adaptivity refers to the capacity of organisms to differentially evaluate encounters with the environment based on their consequences for the organism’s self-individuation (Varela, 1991). The organism *should* effect action on environmental flows of matter and energy when they contribute positively to the organism’s sustaining of its viability. When an event threatens to destabilize an organism’s capacity for self-individuation it *should* take compensatory action. Conditions of viability are here providing the values relative to which interaction with the environment can be regulated. Viability is a source of value because the organism has an interest in its own continued existence. See Kiverstein and Rietveld (2018) for further discussion of how the enactive idea of adaptivity relates to the ecological notion of situated normativity.

12 Our notion of bodily normativity was primarily inspired by Canguilhem and Merleau-Ponty, but can be related to what is sometimes called “sense-making” in the enactive literature. The organism relates to its surroundings based on a concern for its own continued existence as an individual. The regulation of its activities based on this concern is what enactivists call “sense-making.” The environment of an organism is not a value-neutral space in which it acts but is imbued with what Varela (1997) described as a “surplus of significance.” It is “a place of valence, of attraction and repulsion, approach or escape” (Thompson, 2007, p. 158). Sense-making, is “viable conduct” because the environment’s significance (its “sense”) is enacted by organisms in their active engagement with the environment. The classic example is the chemotaxis of the bacteria in which the tumbling or swimming forward of the bacteria depends on what it senses, but what it senses depends on how it moves. This sensorimotor loop is an example of sense-making in the enactive tradition.

13 The expression “single complex sensitivity” is due to McDowell’s (1998) work on Aristotle. He uses the expression to describe the second nature the skilled individual (or *phronimos*) cultivates that allows them to appreciate what the right thing to do is in a particular situation.

14 More precisely, we define the affordances of the human environment as relations between aspects of the sociomaterial environment in flux, and abilities available in a form of life (see Rietveld and Kiverstein, 2014). We borrow the notion of “forms of life” from Wittgenstein (1953) who used the term to refer to regular, and relatively stable patterns of activity that can be observed in the social world. We argue that the affordances of the human environment grow out of the regular activities of people as they engage in practices. It follows that the environment people act in isn’t only a material environment – it is sociomaterial because the materials people make use of in their activities have been organized and structured through a history of practices.

15 A reviewer notes that whether or not a person experiences their environment through their pathological embodiment will most likely vary a great deal across individuals. It will depend on factors such as personality, level of social support, level of energy in a particular situation, self-confidence and self-esteem and so on. We fully agree with the reviewer that pathological embodiment is likely to be a consequence of the person’s past history and their intersubjective relation with other people. We are grateful to our reviewer for discussion of this point.

16 We note that in making this claim we do not mean to medicalize Michael’s condition. A central problem with the medical model of disability is that it conceives of pathology in terms of a defective body to be treated and cured through rehabilitation or normalization. We agree with Barnes (2016) that “to be physically disabled is not to have a defective body” (p. 1). At the same time we suggest it is important to do justice to the vulnerability and suffering that can be part of living with a physical disability. By understanding experiences of pathological embodiment in terms of catastrophic reactions we think we can do justice to both of these points. Our concept of pathological embodiment is developed to do justice to the very real difference in lived experience that are characteristic of illness. We avoid medicalizing disability but we also seek to do justice to the differences in lived experience that we will see, through reflection on the case of Michael, can be a part of living with CP.

17 One might object that Michael’s experience of his embodiment can only be described as pathological if one endorses our rather idiosyncratic, and non-standard understanding of pathological embodiment. In order to justify this description of Michael, we would therefore need to say more in defense of our understanding of normality and pathology. But recall that our account of pathological embodiment follows from the EE account of bodily normativity outlined in section “The Embodiment of Disability.” Our argument for this account is that it is developed to account for the lived experience of persons, and the way in which the body isn’t simply something a person possesses but is the lived medium of their experience of the world. We suggest such an argument is further supported by the reports from people living with CP we have given above, and in the rest of the article.

18 Gallagher (2018) introduces the concept of an affordance space which he tells us is “the full range of possible affordance fields relative to an individual, including the current affordance field plus any possible changes in that field due to changes in physical or cognitive skills or environment” (p.722; c.f. Brincker, 2014). Gallagher’s affordance space concept overlaps our distinction between the field and landscape of affordances. It can be compared to the field insofar as it concerns relevant affordances in relation to the individual agent over time. But it can also be compared to the landscape of affordances in relation to an individual insofar as it concerns the set of *possible* fields for an individual and this goes beyond those multiple affordances that are soliciting action at a given moment. Gallagher suggests the affordance space for a disabled person that uses a wheelchair will differ from that of a non-disabled person. Their affordance space will for instance depend on their wheelchair skills, the layout of the environment, and the social and cultural attitudes of others in ways that the affordance space of the non-disabled person does not (ibid, p.723). We agree with Gallagher there will be differences in how a disabled person engages with the landscape of affordances. A person that is born blind will not be able to drive a car for instance. We suggest that we can account for this difference in what Gallagher calls the affordance space of the disabled persons in terms of the different skills and abilities disabled people develop. The blind person’s lack of access to the affordances of driving is due to their inability to drive, both now and in the future. Crucially we are arguing the other skills they develop will often allow them to access unconventional affordances available in the landscape. They are still able to establish new and better ways of doing things in many cases and so their disability doesn’t necessarily entail a pathological embodiment. Thanks to the reviewer for drawing our attention to Gallagher’s (2018) paper and the relevant example.

19 The conviction of being capable is not, by itself, enough for someone to be normally embodied. The person might be anosognosic, or pathologically grandiose in their estimation of their own capabilities. They may well think they can do things they actually can’t. Our thanks to the reviewer for pressing us on this point. We have provided an analysis of normal embodiment in terms of a person being able to establish a dynamic stability with the multiple affordances that are relevant to them. Our account of normal embodiment is not dependent on what a person believes they can do but on whether they are able to tend toward an improved grip on a field of relevant affordances.

20 Patterson and Hughes have described the experience of I-cannot using Drew Leder’s notion of “dysappearance” (Leder, 1990). The disabled body dysappears in the sense that it becomes the focus of attention. Typically in lived experience the body recedes into the background - it is the point of view from which we engage with the world. The body obtrudes into lived experience when a person is in pain but also in disability due to the hostile environment the disabled person lives in. As Patterson and Hughes write: “When one is confronted by social and physical inaccessibility one is simultaneously confronted by oneself. When one encounters prejudice in behavior or attitude, one’s impaired body dysappears.” (Paterson and Hughes, 1999: p.603).

21 See Martiny (2015) for a more detailed analysis of the need for “constant adjustment” experienced by people with CP.

22 One might object that the elderly fit our description of pathological embodiment – they structure their environment so as to keep things as constant as possible in order to avoid situations to which they cannot adapt. Yet this is an adequate response to the increasing limitations they experience due to old age rather than a consequence of pathological embodiment. We suggest the crucial difference is that the elderly are driven to avoid change not in order to avoid catastrophic reactions in which they fail to adapt to the environment. What they do is maintain themselves in situations they are able to manage well just as a part of their skilled engagement with the environment. Shrinking the space of affordances they open themselves to is what it takes for them to skillfully tend toward an optimal grip.
